# Oil/Water Biphasic Solvent System for the Eco-Extraction and Cosmetic Formulation of *Bixa orellana* L.

**DOI:** 10.3390/plants13141940

**Published:** 2024-07-15

**Authors:** Marine Chambaud, Ariane Fournier, Clément De Saint Jores, Benjamin Caux, Cyril Colas, Emilie Destandau

**Affiliations:** 1 Institut de Chimie Organique et Analytique, Université d’Orléans, CNRS UMR 7311, 45100 Orléans, France; marine.chambaud@univ-orleans.fr (M.C.); clement.de-saint-jores@univ-orleans.fr (C.D.S.J.); benjamin.caux@univ-orleans.fr (B.C.); cyril.colas@univ-orleans.fr (C.C.); 2 Terre de Couleur, 6 rue de Châtenay, 37210 Rochecorbon, France; 3 Interfaces, Confinement, Matériaux et Nanostructures, Université d’Orléans, CNRS UMR 7374, 45100 Orléans, France; ariane.fournier@cnrs-orleans.fr; 4 Centre de Biophysique Moléculaire, CNRS UPR 4301, 45071 Orléans, France

**Keywords:** eco-extraction, achiote, water, UHPLC-HRMS, cosmetics

## Abstract

Annatto, obtained from the seeds of achiote (*Bixa orellana* L.), is a widely used orange pigment rich in bixin and other apocarotenoids. This work reports the optimisation of a green extraction method of pigments and antioxidant compounds from achiote as well as its integration in a one-step green extraction-cosmetic formulation process. A biphasic solvent system of water and oil was used to recover simultaneously polar polyphenols, and less polar compounds, such as δ-tocotrienol and bixin. The optimisation of the ultrasound assisted extraction is presented, as well as a comparison of different vegetable oils used as extraction solvents. The composition, physicochemical properties and antioxidant activity of the oils were studied and their extraction performance was compared. Refined sunflower oil proved to be a better solvent than virgin olive, jojoba, coconut and grapeseed oils. Both aqueous and oil phases displayed an interesting antioxidant capacity. The oil phase contained 0.9% of bixin, as well as minor apocarotenoids and δ-tocotrienol. Twelve compounds, mainly phenolics, were identified by UHPLC-DAD-HRMS/MS in the aqueous phase. Twenty-one volatile compounds were identified in the volatile fraction by SPME-GC-MS. Lastly, a one-step green process is proposed to combine the extraction and the cosmetic formulation of the bioactive compounds.

## 1. Introduction

*Bixa orellana* L., commonly known as achiote, is a Brazilian tree commonly grown in tropical areas. It was nicknamed “lipstick tree” because of the red-orange pigment called annatto extracted from its seeds and arils. The Aztec people used annatto to colour their bodies, clothes and food and it is still one of the main natural pigments used today in the food, cosmetic and pharmaceutical industries. Moreover, the seeds of *Bixa orellana* are part of South American traditional medicine to treat various illnesses, including burns and wounds [1,2]. The main compounds in achiote seeds are apocarotenoids, in particular bixin and norbixin, which are responsible for the orange colour of the extracts. Apocarotenoids are also potent antioxidants with photoprotective and anti-inflammatory properties that have been exploited in the cosmetic field in UV-filtering and skincare products [1,3,4,5]. However, Cardarelli et al. found a correlation between total phenolic content and free radical scavenging ability, highlighting the role of polyphenols in the antioxidant activity of *Bixa orellana* extracts [6]. Few studies have focused on the characterisation of polyphenols from achiote seeds and only a few chalcones, flavonoids and phenolic acids have been described [7,8]. Apart from apocarotenoids, the main compounds described from achiote seeds are tocotrienols, as well as sesquiterpenes and monoterpenes in the volatile fraction [2]. Finally, other compounds such as tannins and saponins have been detected by colorimetric assays, but never identified [9]. All these compounds are known in cosmetics for their antioxidant, anti-ageing, or antibacterial properties [10,11]. 

Given the wide range of polarity of these molecules, there is no single solvent able to extract them all simultaneously. Polar phenolic acids are generally extracted using hydroalcoholic solvents while less polar compounds such as apocarotenoids or tocotrienols are generally recovered using organic solvents such as ethyl acetate or hexane, or vegetable oils [1,12,13]. To extract simultaneously all these compounds, an eco-extraction using a biphasic solvent system of vegetable oil and water was developed.

Green extraction is a concept derived from green chemistry with the same objective of developing more sustainable processes [14,15]. The six objectives of green extraction aim at achieving an economy of steps, solvents and energy, sustainable sourcing and reduction of waste, and the obtention of biodegradable safe extracts [14]. Oil and water are both accessible green solvents commonly used in cosmetics [16]. Water is able to extract polar molecules, including potential bioactives such as phenolic compounds. As water is the main ingredient in most cosmetic emulsions, it could be interesting to use an aqueous extract, enriched in active compounds, instead of plain water in formulation. Vegetable oil is a bio-based solvent that is conventionally used for the extraction of bixin and is efficient for the extraction of carotenoids and essential oils [2,17]. In the cosmetic field, oil is not only the main component of the fatty phase of the formula, but also a potential active ingredient with emollient and antioxidant properties. Moreover, compounds dispersed in oil are less prone to oxidative damage [17], as was shown for bixin by Kanjilal and Singh [18]. 

Ultrasound assisted extraction (UAE) was chosen to lower the viscosity of oil and increase exchanges at the interface between phases, thus facilitating the extraction of achiote in both solvents through acoustic cavitation [19,20]. UAE has already been described as efficient for the extraction of carotenoids from various plants, including for the extraction of bixin from achiote [8,20]. It is considered a green extraction technique as it allows a similar or better yield than conventional extraction methods in less time and at lower temperature [21]. Thus, it can also protect apocarotenoids from thermal degradation [8]. A water/sunflower oil biphasic solvent was used as a model to optimise the extraction parameters (duration of extraction, ultrasound frequency and plant/solvent ratio) to obtain a high content in polyphenols and apocarotenoids. 

Some studies have shown that the oil used as a solvent can impact the extraction of volatile compounds, polyphenols or carotenoids [22,23,24]. This is due to different physicochemical properties, such as the viscosity or the oil/water interfacial tension as well as to the composition both in fatty acids and minor compounds of the oils, such as tocopherols or phenolic compounds [17,24]. Thus, oils with different compositions were evaluated to determine their influence on the composition and antioxidant activity of the oil phase, but also of the aqueous phase. Biostatistics tools applied to UHPLC-HRMS analyses were used to compare in depth the aqueous phases and assess the influence of the oils. Sunflower oil used for parameter optimisation was compared to coconut oil, grapeseed oil, virgin olive oil and jojoba oil, which all possess different compositions and are widely used in cosmetic formulations. The physicochemical properties of the oils, as well as their fatty acid content were also obtained to discuss the results. The optimised extract was characterised by UHPLC-HRMS/MS and SPME-GC-MS. It was then formulated in an oil-in-water (O/W) emulsion. Finally, a combined ultrasound-assisted extraction and formulation was performed. This methodology allows a green one-step process from the plant to a multifunctional potent cosmetic product.

## 2. Results and Discussion

### 2.1. Selection of Ultrasound-Assisted Extraction parameters

As described in the literature, achiote contains both polar and non-polar compounds, such as phenolic compounds or apocarotenoids, of interest for cosmetic applications [2,4,6]. Thus, a biphasic oil/water extraction was developed to recover simultaneously all compounds from achiote. Oil was used to extract apocarotenoids and tocotrienols, while water was used to obtain other antioxidant compounds such as phenolic compounds. The biphasic ultrasound-assisted extraction was first developed using an accessible oil containing no amphiphilic compounds, namely refined sunflower oil. 

To develop the ultrasound-assisted extraction, three parameters were studied: ultrasound frequency, time and plant-to-solvent ratio (Table S1 in Supplementary Data). The ultrasound frequency was the only parameter that could be controlled on the ultrasound reactor. Moreover, it is known to greatly impact the extraction efficiency [25]. Three values were evaluated 5, 13.75 and 22.5 kHz, corresponding to 10, 40 and 100% of the reactor capacity. For the duration of extraction three extraction times 10 min, 30 min and 60 min were assessed to cover a wide time range while avoiding overheating. Indeed, the temperature was directly linked to ultrasound frequency and time. As the reactor was not thermostated, an increase of temperature up to 80 °C was observed for 60 min of extraction. The plant-to-solvent ratio (0.1, 0.55 and 1 g/20 mL) was chosen to obtain even for the higher ratio a total immersion of plant matrix in the extraction solvent that favor exchanges between the plant and the two phases. To assess the repeatability of the extraction process, six extracts were made at 35 min, 13.75 kHz and 0.55 g/20 mL. 

The oil phase and the aqueous phase were separated and both analysed. The UV-visible spectrum between 350 and 550 nm of the oil phase was characteristic of its apocarotenoid content with three main absorption bands at 430, 465 and 486 nm. Hence, the colour of the oil phase, represented by its absorbance at 465 nm (*λ*_max_ of the extract) was used to compare the extracts. The absorbance at 465 nm of the oil phases after a ten-fold dilution in sunflower oil ranged between 0.22 and 2.32 a.u. The 6 replicates had an average absorbance of 1.11 ± 0.28 a.u., showing an acceptable repeatability of extraction and analysis despite the viscosity of the oil (Table S1 in Supplementary Data).

The aqueous phase was a potential source of polyphenols, a large family of secondary metabolites often used in cosmetics for their well-established antioxidant activity [26]. Indeed, studies have pointed out the richness of achiote in multiple families of polyphenols such as phenolic acids, chalcones, flavonoids or tannins [9]. Therefore, the total polyphenol content (TPC) of the aqueous phase was measured. The TPC of the aqueous phases ranged between 0.02 and 0.22 mg of gallic acid (GA) equivalent/mL. The average TPC of the 6 replicates was 0.14 ± 0.02 mg GA eq./mL, showing once again a good repeatability of both the extraction process and the analysis (Table S1 in Supplementary Data). 

The extract that displayed the brightest orange colour, with an absorbance at 2.32 ± 0.23 a.u., and the highest content in polyphenols at 0.22 ± 0.01 mg of GA eq./mL or 2 mg of GA eq./g of achiote powder was obtained with 1 g of achiote/20 mL of solvent at 22.5 kHz for 35 min. The quantification of the apocarotenoid content in this extract revealed that there was 0.9% of bixin equivalent in the best extract. The maximum possible concentration of bixin in oil being 1%, this results shows that the extraction process is efficient to recover the pigment from achiote [2].

### 2.2. Oil Selection

Studies have shown that different oils have different extraction capabilities based not only on their fatty acid, but also on their content in minor compounds such as tocopherols or phenolic compounds [17]. Both unsaturated fatty acids and amphiphilic compounds in the oil can potentially increase the extraction yield of compounds with medium polarity such as bixin [22]. To evaluate the influence of the oil type on the biphasic extraction five vegetable oils with different properties were selected: refined sunflower oil, coconut oil, jojoba oil, virgin olive oil and grapeseed oil. To compare the properties of these oils, their composition was assessed by supercritical fluid chromatography coupled to mass spectrometry (SFC-MS). The density, viscosity and interfacial tension of the oils were also measured as well as their antioxidant activity. 

Then, the extraction of achiote was performed in triplicate with each oil. The influence of oil was evaluated not only on the oil phase, but also on the aqueous phase since minor polar compounds of the oil could be partitioned between both phases and potentially influence the aqueous phase extraction capacity and its final composition. To do so, the colour of the oil phase and the antioxidant activity of both phases were measured. The same antioxidant activity assay could not be used on both phases of the extract because of miscibility and precipitation issues. However, the two assays used, DPPH and ABTS, share a similar mechanism of action and both assess the radical scavenging capacity [27]. Finally, the composition of all aqueous phases was analysed by UHPLC-HRMS to enable comparison using a statistical approach.

#### 2.2.1. Characterisation of Crude Oils

The fatty acid content of the oils was analysed by SFC-MS (Table 1). Sunflower, olive and grapeseed oils were mainly composed of C18 fatty acids such as oleic (O, C18:1) and linoleic (L, C18:2) acids, as well as palmitic acid (P, C16:0) for olive oil, as described in the literature [28,29,30]. Indeed, almost 50% of the triglycerides identified from sunflower and grapeseed oils were LLL and LLO, while the main triglycerides of olive oil were OOO and POO. 

The analysis of coconut oil highlighted a large majority of triglycerides composed of saturated fatty acids (SFAs). Moreover, 83% acylglycerols contained lauric acid (La, C12:0), a saturated fatty acid reported as the main fatty acid from coconut oil [31]. Triglycerides from coconut oil eluted around the same retention time as diglycerides from grapeseed, olive and sunflower oils. This is because they contain mainly short and medium-chain fatty acids (respectively C2–C6 and C8-C12) while the other oils contain mostly long-chain fatty acids (C14–C24) [31].

Jojoba oil is a liquid wax and contains mostly apolar esters of long-chain fatty acids (C20 and C22) [32]. The SFC-MS analysis of jojoba oil gave four peaks, identified as eicosenyl oleate (C18:1/C’20:1), eicosenyl eicosenoate (C20:1/C’20:1), docosenyl eicosenoate (C20:1/C’22:1) and/or eicosenyl docosenoate (C22:1/C’20:1) and docosenyl docosenoate (C22:1/C’22:1) and/or tetracosenyl eicosenoate (C20:1/C’24:1) [32,33]. The chromatographic profile and the proportions of compounds were in accordance with results from Busson-Breysse and colleagues [33].

The physicochemical properties of the oils potentially relevant to the extraction efficiency were also assessed (Table 2). Generally, a low viscosity is associated with better extraction capacity because it allows a better penetration of the solvent in the solid matrix to reach the molecules and a better diffusivity of compounds in the solvent [22,24]. Jojoba oil had the lowest viscosity at 32.8 mPa.s, and olive oil the highest one at 54.5 mPa.s. Sunflower oil was the second most viscous oil at 50.2 mPa.s and coconut and grapeseed oil had an intermediary viscosity respectively at 40.8 mPa.s and 43.9 mPa.s. Another factor to consider is the oil/water interfacial tension as it could affect the solubility of apocarotenoids as well as the diffusivity of compounds between phases [34]. Indeed, the polarity of an oil affect its interfacial tension with water: the higher the polarity, the lower the interfacial tension [34]. Coconut oil had the lowest interfacial tension (12.5 mN/m) while jojoba oil had the highest (24.6 mN/m), meaning that coconut oil was the most polar oil tested while jojoba oil was the least polar. These results are in agreement with the oil compositions observed using the SFC-MS analysis.

#### 2.2.2. Comparison of Oil Phases

**Figure 1:**
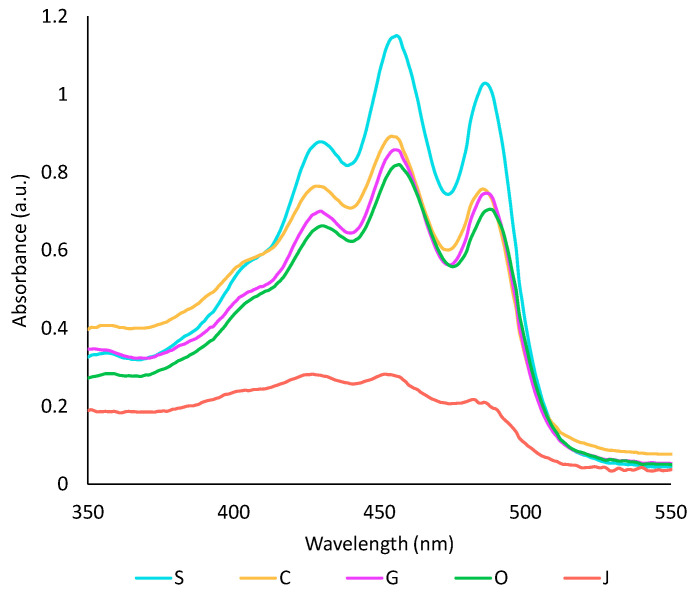
Visible spectra of oil phases after a ten-fold dilution in heptane. S: sunflower oil, C: coconut oil, G: grapeseed oil, O: olive oil, J: jojoba oil.

The capacity of the five studied oils to extract pigments and antioxidant compounds from achiote was assessed. First, the colour of all oil phases was compared by UV-visible spectrophotometry (Figure 1). Sunflower oil extract showed the highest absorbance in the visible region, while jojoba oil had the lowest one. These results can be directly correlated to the oil’s capacity to extract carotenoids. From previous studies, oils with shorter chain-length fatty acids tend to be more efficient for the extraction of carotenoids, while the degree of unsaturation has no impact [35,36]. Thus, the low yield of carotenoids obtained with jojoba oil was most likely caused by its high content in long-chain fatty acids. In the literature, virgin oils tend to give higher yields because they contain amphiphilic compounds [17,37]. However, Chutia and Mahanta showed that the extraction temperature influences the extraction of carotenoids in olive oil, but not in sunflower oil. They concluded that sunflower oil may be less prone to thermal degradation than olive oil, leading to fewer changes in the composition of the oil [35]. This could explain why refined oils, particularly sunflower oil, were more efficient than olive oil in the present study. Some parameters such as refining method, water content in the oil, oil quality or interaction with the aqueous phase may also have impacted the extraction. 

The extraction was optimised to obtain the richest colour, but also the highest antioxidant activity since an antioxidant extract is interesting in cosmetics both to improve the stability of the product and to protect the skin against oxidative damages from the sun, age or pollution [26]. The antioxidant activity was investigated both in the crude oil and in the two phases of the extracts. 

**Figure 2:**
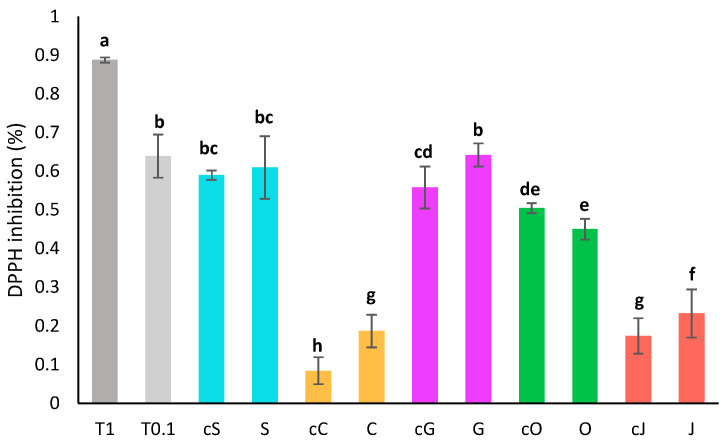
Antioxidant activity by DPPH assay of oil phases diluted twofold compared to the activity of Trolox at 1 mg/mL (T 1) and 0.1 mg/mL (T 0.1). S, C, G, O and J: oil phases obtained respectively with sunflower, coconut, grapeseed, olive and jojoba oil. cS, cC, cG, cO and cJ: corresponding crude oils. Groups a–h are significantly different based on the analysis of variance (*p* < 0.05).

The antioxidant activity of oils before extraction and of oil phases was assessed by DPPH assay after a two-fold dilution in heptane (Figure 2). The pure oils displayed a wide range of antioxidant capacity from 8 ± 4% of inhibition for the diluted coconut oil to 59 ± 1% of inhibition for the sunflower oil. Sunflower and grapeseed oils displayed the best radical scavenging capacity, while coconut and jojoba oil showed the lowest DPPH inhibition. DPPH inhibition thus seemed correlated with the amount of unsaturated fatty acids. A similar correlation was previously described by Sabudak et al. on oils of the *Trifolium* species [38]. 

The activity of the extracts was slightly higher than that of the pure oils, especially for the oils that had a low activity to begin with. This is most likely due to the content in carotenoids, which are powerful antioxidants [4]. Olive extract is the only exception, potentially because a portion of the polyphenols from the oil were degraded as suggested earlier or were partitioned in the aqueous phase during the extraction process.

#### 2.2.3. Impact of the Oil on the Aqueous Phase

**Figure 3:**
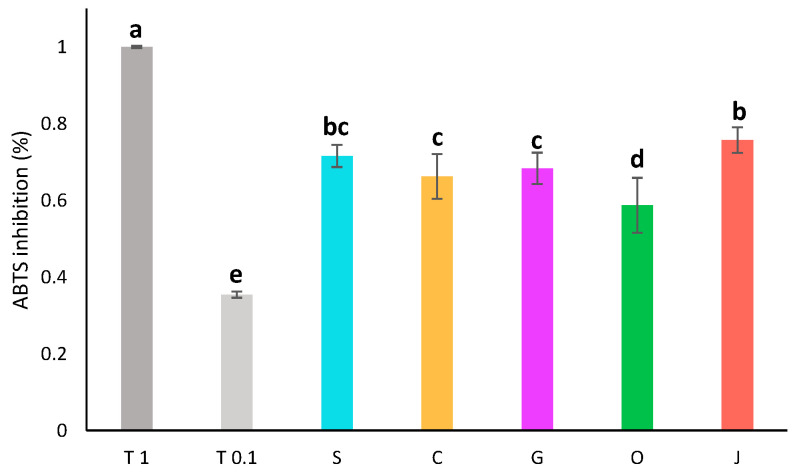
Antioxidant activity by ABTS assay of aqueous phases diluted twofold compared to the activity of trolox at 1 mg/mL (T 1) and 0.1 mg/mL (T 0.1). S: sunflower oil, C: coconut oil, G: grapeseed oil, O: olive oil, J: jojoba oil. Groups a–e are significantly different based on the analysis of variance (*p* < 0.05).

The oil used as a solvent had a great impact on the pigment content and antioxidant activity of the final extract. To determine if it also had an impact on the composition or activity of the aqueous phase, the antioxidant activity of the aqueous phases was assessed by ABTS assay (Figure 3). The diluted aqueous extracts all displayed an antioxidant activity around 68 ± 6% of inhibition. Aqueous phases displayed quite similar antioxidant activities. Jojoba and sunflower aqueous phases were slightly more active, while olive aqueous phase had a lower activity.

**Figure 4:**
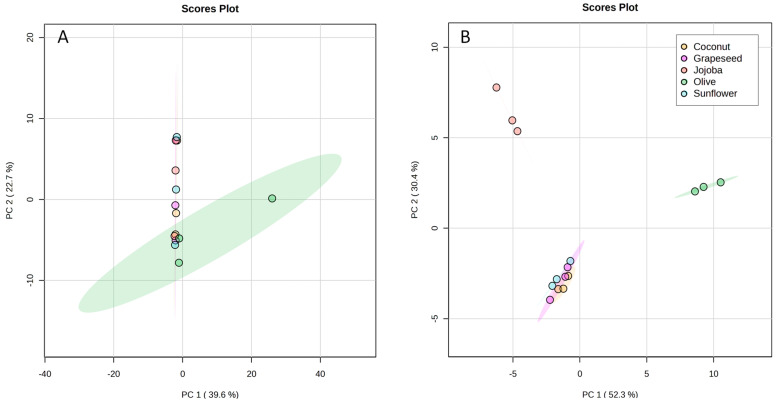
PCA score plots built from (**A**) the total matrix of the UHPLC-HRMS data, (**B**) the reduced matrix of the UHPLC-HRMS data. Coconut in yellow, grapeseed in purple, jojoba in red, olive in green and sunflower in blue.

To assess more finely the differences between aqueous phases, their composition was analysed by UHPLC-HRMS and a statistical analysis was performed. A principal component analysis (PCA) was obtained from the total UHPLC-HRMS data. The first (PC1) and second (PC2) principal components represented only 39.6% and 22.7% respectively of the information (Figure 4A). Thus, this PCA did not allow the discrimination of the extracts. 

Nevertheless, the ANOVA highlighted 91 features that had significantly different intensities amongst the samples. To observe the separation of these features between the samples, the matrix was reduced so that it contained only these significant features and was processed through MetaboAnalyst. The PCA of the reduced matrix (Figure 4B) showed differences between the groups. The quality control (QC) samples (not represented here) were clustered together at the centre of the PCA, showing the good quality of the data. This PCA showed a better representation of the information with PC1 representing 52.3% and PC2 30.4% of the information. Sunflower, coconut, and grapeseed extracts were grouped together, showing that the polarity and the degree of unsaturation of fatty acids did not have a significant effect on the composition of the aqueous phase. Olive and jojoba extracts were separated from the others along PC1 and PC2. This discrimination could be attributed to the type of oil as only the virgin oils showed significant differences. Indeed, virgin oils tend to contain some polar compounds that could have been solubilised in the aqueous phase. Olive oil is known for its high polyphenol content [30], while jojoba oil only contains flavonoids in low concentrations [32].

**Figure 5:**
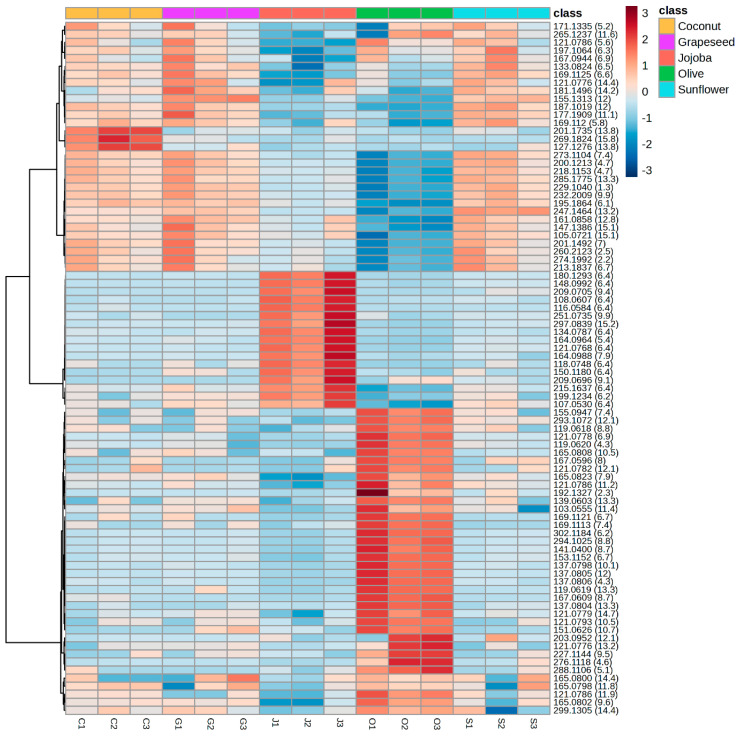
Heatmap highlighting the variation between the samples of the intensity in positive ionsiation of the 91 features from the reduced matrix. The scale ranges from blue (lower intensity) to red (higher intensity). Compounds are organised by similarity of intensity in the samples. Three independent replicates of the aqueous phase were analysed for each oil.

To understand the differences between the groups, a heatmap was constructed from the same data (Figure 5). The 91 significant features of the reduced matrix covered a large polarity range, with retention times between 2.2 and 15.8 min. Coconut, grapeseed and sunflower extracts had similar profiles and their main features were less intense in jojoba and olive extract. This result explains how coconut, grapeseed and sunflower were grouped in the PCA. The heatmap also highlighted features specific to jojoba extracts and olive extracts. From the 91 features in the reduced matrix, 17 were significantly more intense in the jojoba aqueous phase and 38 were significantly more intense in the olive aqueous phase, showing that these features were specific to the extracts obtained with those oils. While those features were mainly representative of jojoba or olive extracts, they were still present in the other extracts in significantly lower concentrations. 

This heatmap suggests that these features are related to compounds of achiote rather than compounds coming from the oil solubilised in the aqueous phase. However, the chromatographic profiles of all aqueous phases were similar, meaning that these features corresponded to minor compounds or their fragments. This was especially the case for jojoba oil as 9 of the significant features corresponded to fragments of a single compound eluted at 6.4 min. The main molecules were likely excluded during the reduction of the matrix as they were not significant features and did not allow the differentiation of the groups. The low concentration of specific molecules explains why all extracts have such similar antioxidant activities. 

Based on all these combined results, sunflower oil was found to be the most suitable oil among the ones tested. Sunflower extracts displayed the brightest orange colour and were amongst the most antioxidant for both the oil phase and the aqueous phase. Thus, the optimised extract used for the rest of the study corresponds to a 35 min extraction at 22.5 kHz, using 1 g of plant for 20 mL of solvent (water/sunflower oil, 50/50).

### 2.3. Characterisation of the Optimised Extract

#### 2.3.1. Characterisation of the Oil Phase

Oil extracts from achiote seeds are commonly used as food dyes in which bixin represents more than 80% of the pigment content [1]. Non-polar extracts of *Bixa orellana* are also rich in tocotrienols, mostly δ-tocotrienol. In fact, achiote seeds are one of the vegetable sources with the highest content in tocotrienols as the seeds of most higher plants contain tocopherols instead of tocotrienols [2]. The optimised oil-phase was analysed by UHPLC-HRMS/MS to determine if the extraction process allowed the recovery of these characteristic compounds. This analysis also allowed the characterisation of the pigment content. Before the analysis, a liquid-liquid extraction (LLE) was performed using MeOH to recover compounds of interest from the oil. The LLE extracts obtained from sunflower crude oil and from the optimised oil phase were then injected in UHPLC-HRMS. The pigment content was detected at 465 nm and identified from the MS and MS/MS data after comparison to the literature and Carotenoid Database. 

The main pigment eluted at 9.77 min, was detected at *m/z* 395.2220 and *λ*_max_ = 432, 459, 486 nm which both correspond to bixin [7]. It represented more than 90% of relative area at 450 nm. Its fragmentation spectrum was consistent with bixin with characteristic fragments attributed to the loss of H_2_O (*m/z* 377.2109) and to the consecutive losses of CH_3_OH (*m/z* 363.1943), CO (*m/z* 335.1907), and H_2_O (*m/z* 317.1907) [7].

The chromatogram at 280 nm revealed one compound in the extract that was absent from the oil. This compound eluted a 11.60 min and was detected at *m/z* 397.3105 and *λ*_max_ = 295 nm, which both correspond to δ-tocotrienol [7]. The fragmentation pattern confirmed the identification with fragments detected at *m/z* 191.1061, 177.0906 and 137.0595 and previously described in the literature [7].

#### 2.3.2. Characterisation of the Aqueous Phase

**Figure 6:**
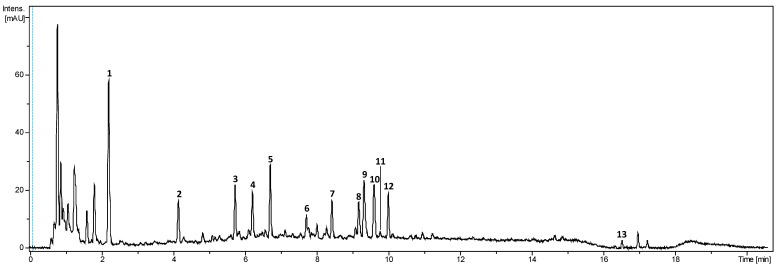
UHPLC-DAD chromatogram of the aqueous phase from the optimised extract cumulated at 280 and 450 nm.

The literature on *Bixa orellana* focuses mostly on the pigments or volatile content. In the few existing studies on the aqueous extracts of achiote seed, different families of compounds are generally detected without further characterisation [9]. The main compounds from the aqueous phase of the biphasic extract were identified using UHPLC-DAD-HRMS/MS. Compound identification focused on compounds also detected by the DAD (280, 465 nm) (Figure 6). Molecular formulae were proposed with a 5 ppm mass tolerance for MS and 10 ppm for MS/MS. The [M+Na]^+^ and [M+NH_4_]^+^ adduct ions and the comparison of positive and negative ionisation modes were used to corroborate the proposed formulae. The literature and public databases (SciFinder, Pubchem and KNapSack) were queried to suggest a putative identification for each compound. The retention time, *m/z*, fragmentation characteristics, *λ*_max_, proposed molecular formula and proposed identification are summarised in Table 3.

Compound **1** was detected in MS^+^ at *m/z* 171.0291 and at *m/z* 169.0138 in MS^-^. The proposed formula was C_7_H_6_O_5_. Fragments were detected in MS/MS^+^ at *m/z* 153.0182 and 127.0388, corresponding respectively to losses of H_2_O (18 u) and CO_2_ (44 u). Fragments derived from *m/z* 153.0120 were also detected at *m/z* 135.0078 and 107.0099, corresponding to the consecutive loss of H_2_O (18 u) and CO (28 u). Lastly, fragment at *m/z* 125.0227 (C_6_H_5_O_3_^+^) and 109.0281 (C_6_H_5_O_2_^+^) were also detected. The molecular formula and the fragmentation pattern corresponds to information previously recorded using our instrument for gallic acid standard and described in litterature, which is also coherent with the *λ*_max_ at 270 nm of the compound [39]. 

Compound **2** was detected in MS^+^ at *m/z* 155.0338 and its proposed formula was C_7_H_76_O_4_. It fragmented into *m/z* 137.0237 after a loss of H_2_O (18 u) and 111.0164 after a loss of CO_2_ (44 u). Both further fragmented into *m/z* 93.0334 (C_6_H_5_O^+^). This pattern of fragmentation has been described in negative ionisation for dihydroxybenzoic acid [40]. According to the literature, *λ*_max_ at 258 and 292 nm most likely corresponds to 2,6-dihydroxybenzoic acid or protocatechuic acid (3,4-dihydroxybenzoic acid) [41]. As the latter is a well-known plant metabolite, compound **2** was putatively identified as protocatechuic acid.

Compound **3** was identified in MS^+^ at *m/z* 445.2069 ([M+H]^+^) as well as at *m/z* 427.1964 ([M+H-H_2_O]^+^) after in source fragmentation. The proposed formula for compound **3** was C_21_H_32_O_10_. Two other fragments were observed at *m/z* 265.1438 and 247.1308 corresponding to the subsequent losses of a hexose (162 u) and H_2_O (18 u). From the MS/MS^+^ data and the UV spectra (*λ*_max_: 268 nm), compound **3** was identified as dihydroxyphaseic acid glucopyranoside, an oxidative metabolite of abscisic acid [42]. Absicic acid is a common phytohormone derived from apocarotenoids [43].

Compound **4** was detected in MS^+^ at *m/z* 293.0302 and its proposed formula was C_13_H_8_O_8_. Fragments were detected at *m/z* 275.0184 and 265.0333 after the losses respectively of H_2_O (18 u) and CO (28 u), and at *m/z* 205.0133 (C_10_H_5_O_5_^+^) and 177.0184 (C_9_H_5_O_4_^+^). Moreover, characteristic fragments of brevifolincarboxylic acid were detected at *m/z* 247.0236 (C_12_H_7_O_6_^+^), 219.0289 (C_11_H_7_O_5_^+^), 191.0339 (C_10_H_7_O_4_^+^) and 163.0390 (C_9_H_7_O_3_^+^) [44]. The UV spectra (*λ*_max_ 276, 357 nm) reinforced the putative identification of compound **4** as brevifolincarboxylic acid [45].

Ellagitannins are hydrolysable tannins formed by esters of galloyl-bis-hexahydroxydiphenoyl (HHDP) with a polyol, generally glucose, and sometimes gallic acid. Their fragmentation can release gallic acid, glucose and HHDP, that is readily converted into ellagic acid [46]. Compound **5** was detected at *m/z* 635.0853 and its proposed formula was C_27_H_22_O_8_. Its fragmentation pattern was characteristic of ellagitannins with a loss of a gallic acid group (170 u) that was detected at *m/z* 465.0663 and a loss of glucose (162 u) that lead to the obtention of ellagic acid, detected at *m/z* 303.0082 (C_14_H_7_O_8_^+^) [46]. Compound **5** was identified as Galloyl-HHDP-glucose.

Compound **6** was detected at *m/z* 383.2067 in positive ionisation and 381.1901 in negative ionisation. Its proposed formula was C_20_H_30_O_7_. In positive ionization, losses of H_2_O (18 u) led to fragments detected at *m/z* 365.1954 and 347.1864. Other fragments were detected at *m/z* 283.1672 (C_19_H_23_O_2_^+^) and 165.0916 (C_10_H_13_O_2_^+^). Formulas were compared to the literature and submitted to public databases (SciFinder, Pubchem and KNapSack), but compound **6** could not be identified.

Compound **7** was detected at *m/z* 303.0142 in positive ionisation and 300.9990 in negative ionisation. Its proposed formula was C_14_H_6_O_8_, and *λ*_max_ 252, 356 nm, which both correspond to ellagic acid. The fragmentation pattern in the negative mode was coherent with the literature with fragments detected at *m/z* 283.9928, 257.0066, 245.0088, 229.0135, 185.0240 and 173.0261 [47].

Compounds **8**–**10** all fragmented into the same ion detected at *m/z* 317.03, which corresponded to the molecular ion of compounds **11** and **12**. The proposed formula for this compound was C_15_H_8_O_8_. Compounds **11** and **12** further fragmented into *m/z* 299.99, which, along with the UV spectra observed, is characteristic of O-methylellagic acid [48] and were thus identified as two different isomers of O-methylellagic acid. Compounds **8** and **9** were detected at *m/z* 396.99 in positive ionization and *m/z* 394.97 in negative ionisation. They fragmented into O-methylellagic acid after the loss of a sulfate groupe (80 u). The proposed formula for these compounds was C_15_H_8_O_11_S and they were identified as sulfate derivatives of O-methyl-ellagic acid. Such compounds have previously been identified in a few plant species, but they are very rare and as such, caution should be taken about the identification of these compounds in achiote [49,50]. Nonetheless, it is not the first occurrence of sulfate derivatives in the plant, as several flavonoids sulfate have already been discovered in the leaves of *Bixa orellana* [2]. Compound **10** was detected at *m/z* 463.0878 and gave methyl-ellagic acid after the loss of a deoxyhexose (146 u). It was identified as a methyl-ellagic acid deoxy-hexose.

Compound **13** was detected at *m/z* 381.2058 with UV spectra characteristic of a carotenoid (*λ*_max_ 429, 455, 485 nm). The proposed formula was C_24_H_28_O_4,_ and it fragmented into *m/z* 363.1950 (C_24_H_27_O_3_) after a loss of H_2_O. From these, compound **13** was identified as norbixin [7]. Norbixin, or orelline, is a hydrophilic demethylated derivative of bixin, and the second main pigment of *Bixa orellana* [2,9].

Most of the identified compounds are phenolic acids or hydrolysable tannins. Both families are known for their interesting activities for cosmetics, with a potential as an active ingredient and as a preservative [51]. Ellagitannins notably, have antioxidant, antibacterial and photo-protective properties [51,52]. Norbixin has been showed to display antioxidant, antibacterial, wound healing and photoprotective activities that can be valorised in cosmetic products [53,54].

#### 2.3.3. SPME-GC-MS of the Volatile Fraction

Achiote has been used traditionally as a spice because of its volatile fraction mainly composed of sesquiterpenes [55]. The optimised total extract was analysed by SPME-GC-MS to characterise its volatile fraction (Table 4). 52 compounds were detected and 21 were putatively identified by comparing the experimental retention index (RI) and mass spectra to data from the NIST 20 database. Ishwarane and spathulenol, previously described as major sesquiterpenes from *Bixa orellana* were identified, as well as isospathulenol and neointermedeol [55,56]. Monoterpenes were also identified, especially α-pinene, which has previously been described as one of the main monoterpenes from achiote, but also sabinene, verbenone, sobrerol and geranyl-acetone [55,56]. Although it was never identified in achiote, geranyl-acetone is an apocarotenoid with a biosynthetic pathway similar to that of bixin [57]. Alkanes and a heterocycle were also putatively identified, though they were derived from the oil rather than from achiote.

### 2.4. Formulation of the Extract

**Figure 7:**
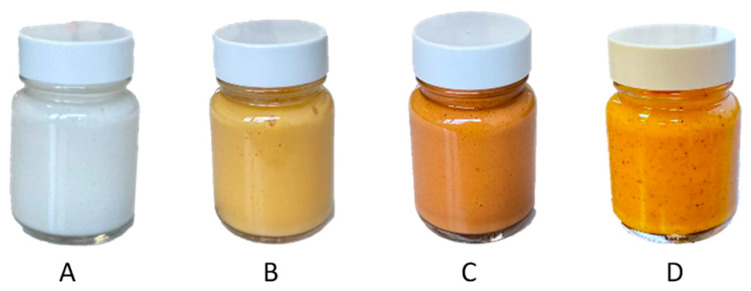
Photograph of the formulations. (**A**): base formulation, (**B**): formulation with the extract, (**C**): formulation with the plant powder, and (**D**): combined extraction and formulation.

**Figure 8:**
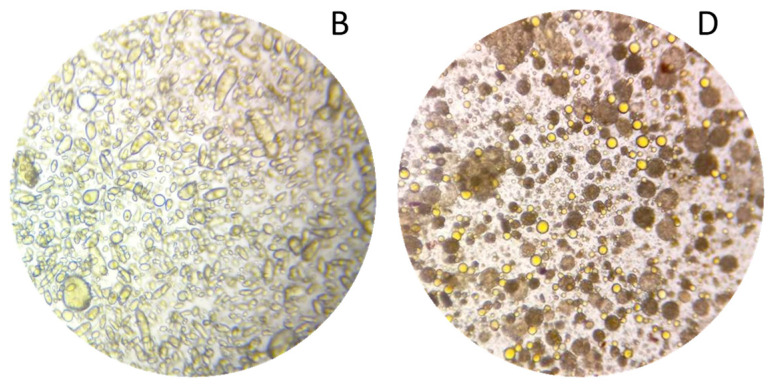
Microscopy at magnification 10× of the formulations (**B**) and (**D**).

The biphasic extraction process allowed the extraction of various compounds known for their interest in cosmetics. To show the potential of this extract for a cosmetic valorisation, it was formulated into a cream. The first step for the formulation of the extract was to create a stable formulation base (**A**). The formulation was formed of three phases, an aqueous phase, a glycerine phase and an oil phase. The phases were first homogenised separately by heating at 65 °C and stirring for 30 min. The emulsion was then formed by mixing the aqueous and glycerine phase and then adding this mixture to the oil phase under constant stirring. From this base, a second formulation (**B**) was prepared by replacing sunflower oil and water by respectively the oil and aqueous phase of the optimised extract. Both emulsions were smooth, creamy, and opaque with a viscosity at 43,940 and 37,044 mPa.s. The base was white, while the formulation with extract was apricot coloured (Figure 7A,B). The microscopic evaluations at magnification 10× of **A** and **B** were very similar, hence only B is shown here (Figure 8B). Both emulsions were stable for 3 months at room temperature and for a week a 42 °C, as well as after a centrifugation test.

Formulation **B** showed that the extract could be implemented in a cosmetic cream as a multi-functional ingredient. However, this method did not allow the full valorisation of the extract as there is only 8.5% of oil in the cream for 78% of water and the method required multiple steps of extraction and formulation. A green process combining the formulation and extraction was developed (**C**) to further enhance the pigment and active compounds content in the final formulation and avoid any waste while reducing the number of steps in the process. To do so, the plant material (1 g/100 g of cream) was directly added during the formulation, half in the aqueous phase and half in the oil phase. The phases were homogenised separately by heating at 65 °C and stirring for 30 min as in the original process, which corresponds to an extraction by maceration. The emulsion was then formed using the same method as for the base formulation. This third emulsion displayed a pumpkin orange colour (Figure 7C) and a higher viscosity around 56,749 mPa.s. It was stable under the same conditions as the previous formulations. The plant particles from the emulsion were separated during the centrifugation, without any dephasing, leaving no residue in the final product.

To intensify the extraction and further reduce the number of steps, a last emulsion was prepared. The phases were homogenised separately with the plant material (1 g/100 g) at 65 °C for 30 min as for emulsion **C**, but the emulsion was performed in only one step. The three phases were directly mixed and subjected to ultrasound, in the optimised extraction conditions (35 min, 22.5 kHz). The emulsion obtained had the same texture quality as the previous ones, as well as a bright orange colour (Figure 7D). It was stable at room temperature and after centrifugation. Unlike the classic emulsion with plant powder, the larger plant particles were already stacked at the bottom of the tube at the end of the process, separated from the emulsion, but the smallest were suspended homogeneously in the emulsion and were both visible and perceptible on the skin (Figure 7D). The microscopic evaluation at a magnification of 10× showed that oil droplets were much smaller in size than in emulsion **B** (Figure 8). Generally, the cream obtained by ultrasound was more fluid and homogeneous than the classic emulsions. Its brighter colour shows that it was also enriched with more pigments than creams **B** and **C**.

This last preparation is a proof of concept for an all-in-one extraction and emulsion using primarily oil and water as solvents. The use of ultrasound intensified the extraction and reduced the size of oil droplets and particles, resulting in a stable emulsion. This green process allows the formulation of potent and colourful cosmetic products while reducing the number of steps and solvent consumption. By using a one-pot method, the formulation was enriched with the target ingredient, bixin, but also with multiple other compounds that add to its potential efficacy and stability. 

## 3. Materials and Methods

### 3.1. Plant Material, Standards and Chemicals

Powdered achiote (*Bixa orellana* L.) seeds came from Cailleau’s herbalist store (Chemillé-en-Anjou, France) and were kindly provided by Terre de Couleur (Rochecorbon, France). Virgin olive oil was purchased from the supermarket (Orléans, France). Sunflower oil, coconut oil, and jojoba oil were obtained from Interchimie (Compans, France). Grapeseed oil came from Tramier (Aix-en-Provence, France). 2,2′-azino-bis(3-ethylbenzothiazoline-6-sulfonic acid) (ABTS) came from Thermo Fischer Scientific (Illkirch-Graffenstaden, France-). Folin-Ciocalteu’s phenol reagent, potassium persulfate, 2,2 Diphenyl 1 picrylhydrazyl (DPPH), 6-hydroxy-2,5,7,8-tetramethylchroman-2-carboxylic acid (trolox) and acetonitrile (ACN) came from Merck (Saint Quentin Fallavier, France). HPLC-grade ethanol absolute, methanol (MeOH), dichloromethane and formic acid were purchased from VWR (Fontenay-sous-Bois, France). Heptane came from Carlo Erba (Val de Reuil, France). Ultrapure water was produced on a Milli-Q IQ 7000 water purification system from Merck Millipore (Darmstadt, Germany). Carbon dioxide (99.7% purity) was provided by Air Liquide (Paris, France). For the formulation, sorbic acid came from Brenntag (Chassieu, France) and glycerine from Interchimie. Xanthan gum (Keltrol^®^) came from CP Kelco (Atlanta, GA, USA), cetearyl Wheat Straw Glycosides and Cetearyl alcohol (Xyliance™) came from Givaudan (Vernier, Switzerland). Cetearyl alcohol (Lanette^®^ O) and glyceryl stearate SE (Cutina GMS-SE) came from BASF (Monheim, Germany). Butyrospermum parkii butter (Shea butter) came from Olvea (Saint Léonard, France).

### 3.2. Biphasic Ultrasound-Assisted Extraction

#### 3.2.1. Optimisation of the Parameters

Ultrasound-assisted extraction was performed using a R.E.U.S. PEX 1N apparatus (Drap, France) operating at 150 W. Twenty milliliters of ultrapure water/vegetable oil (50/50, *v*/*v*) were introduced in a 50 mL centrifuge tube. Sunflower oil was used for the optimisation of parameters as it is easily accessible. The weighed achiote powder was then added shortly before the start of the extraction. The duration of extraction and ultrasound frequency as well as the plant/solvent ratio were optimised simultaneously. Each parameter was tested at 3 different levels corresponding to 10, 35 or 60 min of extraction at 5, 13.75 or 22.5 kHz, using 0.10, 0.55 or 1 g of plant material for 20 mL of extraction solvent. The extraction at the centre point of each parameter (35 min, 13.75 kHz and 550 mg/mL) was repeated 6 times to assess the repeatability. 

The extracts obtained were composed of an upper oil phase and a lower aqueous phase. They were separated by centrifugation using a Jouan Br4i centrifuge (Thermo Scientific, Les Ulis, France) at 12,520× *g* for 3 × 10 min. The aqueous phase was then filtered using a PVDF 0.45 µm syringe filter.

#### 3.2.2. Oil Selection as an Extraction Solvent

Five vegetable oils: refined sunflower oil, coconut oil, virgin jojoba oil, virgin olive oil and refined grapeseed oil, were used to assess the impact of the composition of the oil on the final extract. The extractions were performed in triplicate using the previously optimised parameters (35 min, 22.5 kHz, 1 g/20 mL). The triplicates were performed independently on three separate days. After centrifugation, the phases were recovered separately. 

The impact of the different oils on the colour of the oil phase, the composition of the aqueous phase and the antioxidant activity of both phases was measured. The absorbance spectra of the oil phases were obtained by UV-visible spectrophotometry. The aqueous phases were analysed by UHPLC-HRMS along with a quality control (QC) sample, prepared by pooling 50 µL of the 15 extracts. A statistical analysis of the UHPLC-HRMS data was then realised, using the QC sample to control the analytical repeatability.

### 3.3. Physicochemical Analysis of the Oils

The density of the vegetable oils was determined using a DMA 1001 densitometer (Anton Paar, Les Ulis, France). Temperature was set at 30 °C for coconut oil and 20 °C for all other oils, to match the temperature at which oils were weighed before extraction. All other physicochemical analyses were conducted at 30 °C to be able to compare the oils. 

The viscosity of the vegetable oils was measured on a Kinexus pro rheometer (Malvern Panalytical, Malvern, UK) in rotational mode using a Couette cell at a shear rate ranging from 0.1 s^−1^ to 1000 s^−1^. The experimental results were analysed using a Newtonian model fit to get the dynamic viscosity. 

Oil/water interfacial tension was measured by the pendent drop technique using the Attension Theta tensiometer (Biloin Scientific, Västra Frölunda, Sweden) with the Young-Laplace analyses. The interfacial tension was measured on five drops of each oil and the average of these measurements was calculated, as well as the standard deviation, which was lower than 0.3 mN/m for all measurements.

### 3.4. SFC-MS Analysis of the Oils

The vegetable oils used for the extraction were analysed by SFC-MS. SFC separations were performed on a Nexera UC system from Shimadzu Corporation (Kyoto, Japan) equipped with a CO_2_ pump (LC-30ADSF), a solvent pump (LC-30 CE), an autosampler (SIL-30AC), two column ovens (CTO-20AC), a photodiode array detector (SPD-M20A) and a backpressure regulator (SFC-30A).

Analysis conditions were based on the work of Gros et al. and Lesellier et al. [58,59]. Five octadecyl-bonded silica columns (75 cm of total length), including four Kinetex C18 (150 × 4.6 mm, 2.6 µm) from Phenomenex (Le Pecq, France) and one Accucore C18 (150 × 4.6 mm, 2.6 µm) from Thermo-Scientific (Les Ulis, France) were used in tandem. The column oven temperature was set at 17 °C. Isocratic analyses were performed at 1.6 mL/min during 80 min with 88% of supercritical CO_2_ and 12% of co-solvent (ACN/MeOH 90/10, *v*/*v*). Before injection, the oils were diluted 200-fold in ACN/dichloromethane (50/50). The injection volume was set at 1 µL for all samples. The back-pressure regulator was set at 100 bar and heated at 60 °C to limit the effect of CO_2_ cold depressurisation. 

MS acquisitions were performed on a simple-quadrupole mass spectrometer LCMS 2020 from Shimadzu Corporation with an APCI ionisation source in positive mode. The source temperature was set at 350 °C, the desorption line temperature at 250 °C and the heat block temperature at 200 °C. Internal voltage was set at 4.5 kV. The flow rate of the drying gas was 10 mL/min and the flow rate of the nebulising gas was 1.5 mL/min. No make-up fluid was introduced prior to the MS. Positive scans were performed in the *m/z* value range of 50 to 1000. 

The mass spectra and comparison to literature allowed the identification of most compounds detected. The chromatographic peaks from the Total Ion Chromatogram (TIC) that were putatively identified were integrated and the area percentage of each peak was calculated.

### 3.5. Carotenoid and Phenolic Contents

#### 3.5.1. UV-Visible Spectrophotometry

The colour of the oil phase of each extract was assessed by UV-visible spectrophotometry. The oil phase was diluted tenfold in heptane and 200 µL of the diluted phase was deposited in a 96-well plate. The absorbance spectra between 350 and 550 nm were read on a CLARIOstar PLUS plate reader (BMG Labtech, Champigny sur Marne, France) monitored by the 2.33 MARS data analysis software.

#### 3.5.2. Quantification of Apocarotenoids

The apocarotenoid content was measured in bixin equivalent according to Joint FAO/WHO Expert Committee on Food Additives Monographs [60] modified by Albuquerque and Meireles [61]. The oil phase of the extract was diluted in acetone (twenty-fold) to reach a suitable concentration of pigments for the analysis. The absorbance at 487 nm of the diluted extract was read on a UV-1800 spectrophotometer (Shimadzu, Kyoto, Japan). Bixin content was calculated using the Beer-Lambert law with $E_{1\;\mathrm{cm}}^{1\%}$ = 3090 [60].

#### 3.5.3. Total Phenolic Content Determination

Total phenolic content (TPC) of the aqueous phases was determined using the Folin–Ciocalteu assay in a 96-well plate, as described by Grigoras et al. [62]. A gallic acid standard solution was prepared at 0.6 mg/mL in EtOH and then diluted to obtain solutions ranging from 0.05 to 0.4 mg/mL. First, 20 µL of gallic acid or aqueous phase and 10 µL of Folin–Ciocalteu reagent were deposited in triplicate. The plate was then kept in the dark at room temperature for 8 min before adding 30 µL of aqueous sodium carbonate (20%) and 140 µL of ultrapure water. The plate was incubated for 2 h in the dark at room temperature, then shaken and read at 760 nm on a CLARIOstar PLUS plate reader. One plate was used for each block of extraction (6 extracts analysed per plate). Results are given in mg of gallic acid equivalent/g of extract using a linear equation based on the calibration curves of gallic acid (block 1: y = 36.699x + 0.0539, R^2^ = 0.9987, block 2: y = 35.539x + 0.0585, R^2^ = 0.9981, block 3: y = 36.555x + 0.0629, R^2^ = 0.9976). An ANOVA analysis determined that there was no significant difference at *p* = 0.05 between the slope and origin of the calibration curves from each block.

### 3.6. Determination of the Antioxidant Activity

#### 3.6.1. DPPH Assay

The antioxidant activity of the vegetable oils and the oil phases was assessed by DPPH assay in a 96-well plate, using a method adapted from Lee et al. [63]. Beforehand, the vegetable oils and the oil phases were diluted twofold in heptane. A 0.02 mM DPPH solution and a standard solution of trolox at 1 mg/mL were prepared in ethanol. Then, 10 µL of standard solution, sample or EtOH were deposited in triplicate and 190 µL of DPPH solution were added. The plate was incubated for 30 min in the dark at room temperature. The absorbance was read at 516 nm on a CLARIOstar PLUS plate reader. Results are given in percentage of inhibition of the DPPH absorbance using Equation (1). Trolox, used as a reference, showed 89 ± 1% of inhibition at 1 mg/mL and 64 ± 6% of inhibition 0.1 mg/mL. The absorbance (A) of each sample, standard or blank without DPPH was always subtracted from its absorbance after the reaction with DPPH to account for its absorbance.

(1) $$Inhibition\;\left(\%\right)=\frac{A_{blank}-A_{sample}}{A_{blank}}\times 100,$$

#### 3.6.2. ABTS Assay

The antioxidant activity of the aqueous extracts was assessed by ABTS assay in a 96-well plate, using a protocol modified from Tagliazucchi et al. [64]. The reactant solution was prepared by mixing 2 mL of a 7 mM solution of ABTS and 2 mL of a 2.45 mM solution of potassium persulfate in the dark for 16 h at room temperature. This solution was then diluted in ethanol/water (25/75, *v*/*v*) to reach 20 mL. A standard solution of trolox at 1 mg/mL was prepared in ethanol and the aqueous phases were diluted two- and four-fold in water. Then, 10 µL of the extract, standard or blank were deposited in triplicate and 190 µL of reactant solution were added. The plate was incubated for 30 min in the dark at room temperature. The absorbance was read at 734 nm on a CLARIOstar PLUS plate reader. Results are given in percentage of inhibition of the ABTS absorbance using Equation (1). Trolox, used as a reference showed 100 ± 0% of inhibition at 1 mg/mL and 35 ± 0% of inhibition at 0.1 mg/mL. The absorbance of each sample, standard or blank without ABTS was always subtracted from its absorbance after the reaction with ABTS to account for its absorbance.

### 3.7. Characterisation of Oil Phases

#### 3.7.1. Liquid-Liquid Extraction of the Oil Phase

To allow the UHPLC characterisation of the oil phase, a liquid-liquid extraction (LLE) was performed using methanol as the extraction solvent. Preliminary experiments were done on sunflower oil spiked with α-tocopherol to determine the number of extractions necessary to extract the maximum concentration of compounds of interest. In the end, 1 mL of methanol was used to extract 1 mL of oil phase and the extraction was repeated three times. The 3 mL of methanolic extracts were pooled before injection. The LLE was performed both on the optimised oil phase and on the sunflower crude oil to determine the source of each detected molecule.

#### 3.7.2. UHPLC-DAD-HRMS/MS Analysis

Chromatographic separations were performed on an Ultimate 3000 RSLC system equipped with an autosampler, a binary pump, a thermostated column compartment, and a DAD detector (Thermo Fisher Scientific, Germering, Germany). 

The separation of the oil phase compounds was performed on a Luna Omega C18 (100 × 2.1 mm, 1.6 µm) column (Phenomenex, Le Pecq, France). For all extracts obtained after the LLE of the optimised oil phase and crude sunflower oil, 1 µL was injected and the separation was performed using water (A) and acetonitrile/isopropanol 50/50 (B) in a gradient elution (10–100% B in 11 min). 

MS/MS experiments were performed on a maXis UHR-Q-TOF mass spectrometer (Bruker, Bremen, Germany) with an electrospray ionisation source (ESI), in positive mode. The nebulising gas pressure was set to 2 bar, and the dry gas flow rate was 9.0 L/min at 200 °C. The capillary voltage was set at 4500 V. Mass spectra were summed during 1000 ms in the m/z range 50–1650. All the MS data were processed using DataAnalysis software, version 4.4 (Bruker). Auto MS/MS acquisition was performed in the *m/z* range 50–1650 with 3 precursor ions and the collision energy was set to 25 and 50 eV.

Molecular formulae were generated using the SmartFormula algorithm with an elemental composition of C, H, O and N to an infinite number with a mass accuracy ≤ 5 ppm and were submitted to the SciFinder, PubChem, Carotenoid Database and Knapsack databases and were compared to the achiote literature to propose compound structures.

### 3.8. Characterisation and Comparison of Aqueous Phases

#### 3.8.1. UHPLC-DAD-HRMS/MS Analysis

For the aqueous phases, the stationary phase was a Luna Omega C18 (150 × 2.1 mm, 1.6 µm) column from Phenomenex. The triplicates of aqueous phases obtained with different water/oil systems were injected in randomised order. A blank was injected at the beginning of the sequence and the QC was injected five times throughout the sequence. For each sample, 0.5 µL were injected. A gradient elution (3–45% B in 12 min then 45–90% B in 2 min kept at 90% B for two more minutes before re-equilibrating) was achieved at 0.5 mL/min with water (A) and ACN (B) both acidified with 0.1% formic acid. 

The MS analysis of the samples was performed using the same set of parameters as the oil phases. Full scan MS analysis was performed on all samples. Auto MS/MS acquisition was then used on a QC sample to obtain structural information on major compounds detected by the DAD at 280 and 450 nm. The MS/MS acquisition was performed in positive and negative ionisation modes. In positive mode the acquisition was performed with 3 precursor ions in the *m/z* range 150–1200 with the collision energy set to 15 and 35 eV. In negative mode, the parameters were the same as in positive mode except the capillary voltage was set at 4500 V and the acquisition was performed with 2 precursors with the collision energy set to 35 eV. The same process as for the oil phases was used to generate molecular formulae and propose compound identification.

#### 3.8.2. Statistical Analysis

A statistical analysis was performed to compare the aqueous phases obtained using different vegetable oils. The HRMS data obtained in full scan MS were preprocessed using the software MetaboScape 4.0 (Bruker). A feature table was created, according to the following parameters. The intensity threshold was fixed at 10,000 counts, the minimum peak length was 7 spectra and the minimum peak length (recursive) was 5 spectra. To be included, features need to be present in a minimum of 3 analyses. The retention time range was 0.5 min to 18 min and the mass range was 100–1600. Extracted Ion Chromatograms (EIC) correlation was fixed at 0.8. The matrix was retrieved as a .csv file from this data. It included 5906 variables and 15 samples (3 replicates of the extraction with five different oils) as well as 5 QC samples.

MetaboAnalyst 6.0 was used to perform the statistical analysis of the matrix. For data filtering, features with low repeatability and features that had near-constant intensities between the samples were excluded. This was accomplished by filtering out, respectively: features with a relative standard deviation (RSD) above 20% amongst the QC samples, and the 40% of features that showed the lowest standard deviation (SD) between the samples. 

For the normalisation step, quantile normalisation with a cube-root transformation of data values and a range-scaling were performed. The data were processed through one-way ANOVA to extract the significant features. According to ANOVA, 91 features were significant in the normalised matrix, while 68 features were significant without the normalisation step. 

A new matrix was constructed by combining the two lists of significant features and suppressing duplicates. This reduced matrix contained 91 unique variables. It was processed through MetaboAnalyst 6.0 to perform Principal Component Analysis (PCA), Partial Least Squares—Discriminant Analysis (PLS-DA) and cluster analysis. Median normalisation was applied, with square root transformation and Pareto scaling of the data.

### 3.9. Characterisation of the Volatil Compounds Using GC-MS Analysis

The volatile content of the optimised extract was assessed by GC-MS. The separation was performed on an Agilent (Les Ulis, France) GC 6890N and the MS detection was performed on an Agilent 5973 MSD. Before analysis, the biphasic extract was shaken vigorously to ensure that both the aqueous and the oil phase have an interface with air. Then, 5 g of the extract were transferred in a headspace vial sealed with a septum and an aluminium cap and heated at 60 °C in a water bath for 45 min. A PDMS 100 µm Supelco solid phase micro extraction (SPME) fibre from Merck was then exposed to the headspace of the sample for 30 min, still on the water bath at 60 °C. The SPME fibre was thermally desorbed into the GC injector with a 0.75 mm straight liner. A desorption time of 10 min at 250 °C was performed in splitless mode. The separation was performed on a HP-5 column (30 m × 0.25 mm × 0.25 µm) with a 1 mL/min Helium flow rate using a temperature program (40 °C for 5 min followed by a ramp of 3 °C/min until 200 °C and then 15 °C/min until 250 °C held for 10 min). 

The mass detection was performed in the *m/z* range 30–300.The putative identifications were based on the comparison of mass spectra to the NIST 20 database. The confidence level of hypotheses was increased by comparing the retention index of the compound to the literature. The retention index (RI) of each compound was calculated using an *n*-alkane series from C8 to C20 analysed under the same GC-MS conditions [65]. The relative peak area of each putatively identified compound was calculated to obtain their indicative relative intensity.

### 3.10. Formulation of the Optimised Extract

#### 3.10.1. Base Formulation

The base formulation was an oil-in-water (O/W) emulsion composed of three phases (Table 5) that were prepared separately by heating at 65 °C under constant stirring on a magnetic stirrer hot plate. The aqueous phase (A) was prepared by mixing water (78%) and sorbic acid (0.5%) and then heated to 65 °C. Phase B contained glycerine (3%) and xanthan gum (1%). Phases A and B were mixed under vigorous stirring using an overhead DLS stirrer (VELP Scientifica, Usmate Velate, Italy) at 65 °C. Once homogeneous, the mixture was added to the oil phase (C), obtained by mixing at 65 °C: sunflower oil (8.5%), glyceryl stearate (4%), Xyliance (Cetearyl Wheat Straw Glycosides and Cetearyl alcohol) (2%), cetearyl alcohol (2%) and shea butter (1%). The emulsion was stirred at 65 °C until it presented a uniform appearance and was then gradually cooled down to room temperature. The pH was then adjusted to between 5 and 5.2 with sorbic acid. This base formulation will be named emulsion A in the rest of the article.

Emulsion B was obtained by adding the extract to the base formulation. To do so, water and oil were replaced during the preparation by the aqueous and oil phases respectively. 

#### 3.10.2. Development of an Integrated Extraction-Cosmetic Formulation Process

Two processes using directly achiote powder in the formulation to realise the extraction and formulation in one step were developed. To prepare emulsion C, the three phases were prepared as described for the base formulation, except that 0.5 g of plant powder were added to phase A and C, for a total of 1 g/100 g of total ingredients. Each phase was homogenised for 30 min under heating at 65 °C and stirring on a magnetic stirrer hot plate. Emulsion C corresponded to a maceration performed during the cosmetic formulation.

The last emulsion was prepared in one step and based on the extraction method optimised previously. For this method, the three phases (100 g in total) were directly transferred to a centrifuge tube with 1 g of achiote powder. Extraction and emulsification were performed simultaneously without any other preliminary step, using the optimised ultrasound parameters defined previously (22.5 KHz, 35 min).

#### 3.10.3. Physicochemical Characterisation of the Emulsions

The viscosity of the emulsion was measured on an Alpha rotational viscometer (Fungilab, Sant Feliu de Llobregat, Spain) using the R3 spindle at a shear rate of 1.2236 s^−1^. The emulsion was observed under optical microscopy at magnification 10×. The stability of the O/W emulsion was tested under accelerated conditions by centrifugation (4000 rpm, 15 min) and by placing the samples at 42 °C for a week and at room temperature for three months. The appearance and phase separation were then compared by macroscopic observation to that of the initial emulsion.

## 4. Conclusions

The use of a biphasic solvent system containing vegetable oil and water combined with ultrasound assisted extraction (UAE) allowed the efficient simultaneous extraction of both polar and non-polar compounds from achiote. The extraction was optimised to obtain the highest content in pigments in the oil phase and polyphenols in the aqueous phase. The best extract was obtained after 35 min of extraction at 22.5 kHz for 1 g of plant powder/20 mL of solvent. Five different oils were compared to assess their impact on both the oil phase and the aqueous phase content. Sunflower oil provided the best extraction of pigments and antioxidants in the oil phase. The aqueous phases showed only minimal differences in activity and composition. The statistical analysis of the HRMS data of the different aqueous phases showed some features specific to jojoba and olive oil extracts, but they had no impact on the activity of the extracts and were more likely to come from the plant than from the oil. The UHPLC-HRMS/MS analysis of the sunflower extract allowed the putative identification of 13 compounds in the aqueous phase, including eight tannins and norbixin, a major hydrophilic apocarotenoid of *Bixa orellana*. The oil phase contained mainly δ-tocotrienol and bixin, which represented 90% of the pigment content and 0.9% of the total biphasic extract. The SPME-GC-MS of the volatile fraction allowed the putative identification of 9 monoterpenes and sesquiterpenes, mainly α-pinene and isospathulenol. The extract was successfully formulated in an O/W emulsion that showed no sign of degradation after 3 months. Finally, a one-pot process combining the extraction and the formulation steps was carried out, enabling the formulation of a stable, multi-functional and colourful O/W emulsion in only one step. This process fits with several principles of green chemistry i.e., the use of green solvents (water and oil) and the economy of steps, solvents and energy allowed by ultrasound-assisted extraction.

## Figures and Tables

**Table 1:** Composition of sunflower, grapeseed, olive, coconut and Jojoba oils estimated by SFC-MS.

	Sunflower	Coconut	Grapeseed	Olive	Jojoba
Esters	0.0%	0.0%	0.0%	0.0%	99.2%
Monoglycerides	0.3%	0.4%	0.4%	0.4%	0.8%
Diglycerides	1.1%	1.5%	4.2%	1.8%	0.0%
Triglycerides	98.6%	98.4%	95.3%	97.9%	0.0%
***Triglycerides composition***					
Only SFAs	0.0%	83.3%	0.0%	0.0%	-
SFAs and MUFAs	15.5%	13.2%	7.3%	70.7%	-
≥1 PUFAs	84.5%	3.0%	92.7%	29.3%	-

SFA: saturated fatty acids, MUFA: monounsaturated fatty acids, PUFA: polyunsaturated fatty acids.

**Table 2:** Measured physicochemical properties of the oils.

	Sunflower	Coconut	Grapeseed	Olive	Jojoba
Density	0.92	0.91	0.93	0.91	0.87
Viscosity (mPa.s)	50.2	40.8	43.9	54.5	32.8
Interfacial tension (mN/m)	18.6 ± 0.1	12.5 ± 0.1	22.2 ± 0.2	17.6 ± 0.1	24.6 ± 0.3

**Table 3:** Characterisation of the aqueous phase by UHPLC-DAD-HRMS/MS and putative identification of compounds.

#	t_R_ (min)	Meas. *m/z* ([M+H]^+^)	Error (ppm)	Meas. *m/z* ([M-H]^−^)	Error (ppm)	Molecular Formula	MS/MS^+^ (Intensity)	MS/MS^−^ (Intensity)	λ_max_ (nm)	Putative Identification
1	2.23	171.0291	−1.7	169.0138	2.7	C_7_H_6_O_5_	153.0182 (77309)		270	Gallic acid
							135.0078 (7878)			
							127.0388 (50839)			
							125.0227 (38830)			
							109.0281 (35417)			
							107.0099 (10599)			
2	4.16	155.0338	0.7	153.0185	3	C_7_H_6_O_4_	137.0237 (29126)		258, 292	Protocatechuic acid
							111.0164 17475)			
							93.0334 (3482)			
3	5.75	445.2069	−0.2	443.1921	0.4	C_21_H_32_O_10_	265.1438 (18095)	281.1369 (1045)	266	dihydroxyphaseic acid glucopyranoside
							247.1308 (3248)	263.1292 (500)		
								237.1489 (1975)		
								219.1361 (2263)		
								201.1274 (504)		
								189.1251 (1281)		
								179.0558 (729)		
								161.0446 (1535)		
								119.0352 (1532)		
								113.0244 (2084)		
4	6.22	293.0302	−3.6	291.0133	4.6	C_13_H_8_O_8_	275.0184 (9895)	247.0238 (97693)	276, 357	Brevifolincarboxylic acid
							265.0333 (7850)	219.0280 (16915)		
							247.0236 (44737)	191.0339 (47419)		
							219.0289 (112482)	173.0224 (11496)		
							205.0133 (16005)	163.0394 (6121)		
							191.0339 (35272)	147.0455 (7879)		
							177.0184 (21638)	145.0291 (10327)		
							163.0390 (45035)			
5	6.72	635.0853	4.1	633.0729	0.7	C_27_H_22_O_18_	465.0663 (169038)	463.0499 (110807)	270	Galloyl-HHDP-glucose
							321.024 (65207)	419.0604 (16835)		
							303.0082 (42157)	300.9978 (139141)		
							277.0345 (120888)	275.0182 (46930)		
6	7.73	383.2067	−0.6	381.1901	4.7	C_20_H_30_O_7_	365.1954 (8265)	337.2004 (14030)	285	Unidentified
							347.1864 (2982)			
							283.1672 (2909)			
							165.0916 (2074)			
7	8.44	303.0142	−2.3	300.9990	4.2	C_14_H_6_O_8_	285.0039 (10599)	283.9928 (16311)	252, 295 (sh), 359	Ellagic acid
							275.0208 (15689)	273.0033 (2226)		
							257.0081 (25410)	257.0066 (7373)		
							247.0256 (9045)	245.0088 (8087)		
							229.0133 (12687)	229.0135 (14017)		
							201.0183 (14626)	217.0133 (3027)		
							173.0234 (8093)	201.0159 (8841)		
							145.0282 (3723)	185.0240 (7621)		
								173.0261 (5121)		
								157.0288 (3054)		
								145.0293 (2019)		
								129.0343 (2037)		
8	9.19	396.9862	−0.4	394.9706	-2.0	C_15_H_8_O_11_S	317.0292 (24927)	315.0105 (187933)	248, 359	O-methylellagic acid sulfate
							302.0057 (7488)	299.9902 (190471)		
							257.0080 (2220)			
9	9.33	396.9860	−1.0	394.9701	3.4	C_15_H_8_O_11_S	317.0289 (22803)	315.0105 (120225)	248, 359	O-methylellagic acid sulfate
							284.9989 (1076)	299.9902 (138397)		
							257.0063 (4280)			
							222.1122 (2321)			
10	9.63	463.0878	−1.4	461.0706	4.3	C_21_H_18_O_12_	317.0294 (150666)	315.0132 (95391)	247, 364	methylellagic acid deoxyhexose
							147.0652 (5958)	299.9895 (68927)		
							129.0545 (20771)			
11	9.78	317.0297	−1.6	315.0146	4.1	C_15_H_8_O_8_	302.0055 (11216)	299.9893 (2786)	236, 254, 364	O-methylellagic acid
							246.0156 (2008)			
12	10.01	317.0299	−2.0	315.0133	4.2	C_15_H_8_O_8_	302.0051 (4169)	299.9903 (124255)	235, 250, 366	O-methylellagic acid
							285.0031 (7753)			
							257.0087 (9174)			
							246.0156 (1446)			
							218.0217 (1318)			
							201.0165 (2570)			
13	16.51	381.2058	0.7	-		C_24_H_28_O_4_	363.1950 (1923)		429, 455, 485	Norbixin
							145.1008 (10344)			

**Table 4:** Volatile compounds identified from the SPME-GC-MS analysis of the total optimised extract.

Compound	Match ^a^	Ref. RI ^a^	Exp.RI	Signal Intensity ^b^
Heterocycles				
1,3-bis(1,1-dimethylethyl)-benzene *	859	1249	1246	+
Alkanes				
4-methyl-octane *	863	861	864	+
2-methyl-nonane *	746	964	967	+
Decane *	894	1000	1002	+
2,5-Dimethylnonane *	759	1021	1020	tr
2,6-dimethyl-nonane *	724	1018	1023	+
Undecane *	776	1100	110	+
Dodecane *	892	1200	1200	++
2,6-dimethyl-undecane *	742	1210	1213	+
2,6,11-trimethyldodecane *	770	1275	1274	+
4,6-dimethyldodecane *	773	1325	1321	+
Tetradecane *	827	1400	1400	+
Monoterpenes				
α-pinene	889	937	933	++
Sabinene	845	974	975	+
Verbenone	707	1204	1204	+
Sobrerol	775	1388	1378	+
Geranyl-acetone	771	1453	1446	+
Sesquiterpenes				
Ishwarane	808	1458	1463	+
Spathulenol	841	1576	1571	+
Isospathulenol	907	1638	1623	+++
Neointermedeol	754	1660	1653	+

^a^ Data obtained from the NIST 2020 database, ^b^ relative peak areas (TIC) are indicated as: tr: <1%, +: 1–5%, ++: 5–10%, +++: >10%. * Compounds found in the crude oil as well as in the extract.

**Table 5:** Base formulation of the O/W emulsion.

Phase	INCI Name	Amount (%)	Function
Phase A (aqueous phase)	Aqua	q.s. 100.0	Solvent
	Sorbic acid	0.5	Preservative
Phase B	Glycerine	3.0	Emollient
	Xanthan gum	1.0	Emulsifier
Phase C (oil phase)	*Helianthus annuus* seed oil	8.5	Solvent
	Glyceryl stearate	4.0	Emulsifier
	Cetearyl Wheat Straw Glycosides and Cetearyl alcohol	2.0	Emulsifier
	Cetearyl alcohol	2.0	Emulsifier
	*Butyrospermum parkii* butter	1.0	Viscosity modifier

## Data Availability

The data presented in this study are available on request from the corresponding author. The data are not publicly available due to privacy.

## Supplementary Materials

The following supporting information can be downloaded at: https://www.mdpi.com/article/10.3390/plants13141940/s1, Table S1:Optimisation of extraction parameters using the absorbance of the oil phase and the total phenolic content (TPC) of the aqueous phase.
