# Supplementary material for: Oil/Water Biphasic Solvent System for the Eco-Extraction and Cosmetic Formulation of Bixa orellana L
Source: Plants (Basel). 2024 Jul 15;13(14):1940. doi: 10.3390/plants13141940 (PMC11280842; doi:10.3390/plants13141940)
Supplement: Supplementary file 1 [file plants-13-01940-s001.zip › plants-3063607-supplementary.pdf]

**Supplementary data**

**Table 1.** Optimisation of extraction parameters using the absorbance of the oil phase and the total phenolic content (TPC) of the aqueous phase.

| Extract N° | Time (min) | Frequency (KHz) | Plant/solvent ratio (g/20 mL) | Absorbance at 465 nm (a.u) | TPC (mg GA eq./mL) |
|---|---|---|---|---|---|
| 1 | 35 | 5 | 1 | $0.54 \pm 0.06^{b}$ | $0.14 \pm 0.01^{c}$ |
| 2 | 60 | 13.75 | 0.1 | $0.89 \pm 0.05^{cd}$ | $0.03 \pm 0.00^{a}$ |
| 3 | 10 | 5 | 0.55 | $0.66 \pm 0.07^{bc}$ | $0.06 \pm 0.01^{ab}$ |
| 5 | 35 | 22.5 | 1 | $2.32 \pm 0.23^{h}$ | $0.20 \pm 0.01^{d}$ |
| 7 | 35 | 22.5 | 0.1 | $1.19 \pm 0.07^{ef}$ | $0.03 \pm 0.00^{a}$ |
| 8 | 60 | 13.75 | 1 | $1.32 \pm 0.07^{f}$ | $0.12 \pm 0.00^{bc}$ |
| 10 | 10 | 22.5 | 0.55 | $0.94 \pm 0.07^{de}$ | $0.15 \pm 0.01^{cd}$ |
| 12 | 35 | 5 | 0.1 | $0.22 \pm 0.04^{a}$ | $0.03 \pm 0.00^{a}$ |
| 13 | 10 | 13.75 | 0.1 | $0.43 \pm 0.03^{ab}$ | $0.14 \pm 0.00^{c}$ |
| 14 | 60 | 22.5 | 0.55 | $1.97 \pm 0.12^{g}$ | $0.14 \pm 0.00^{c}$ |
| 15 | 10 | 13.75 | 1 | $0.62 \pm 0.06^{b}$ | $0.06 \pm 0.00^{bc}$ |
| 16 | 60 | 5 | 0.55 | $0.53 \pm 0.08^{b}$ | $0.07 \pm 0.00^{ab}$ |
| CP | 35 | 13.75 | 0.55 | $1.11 \pm 0.28^{ef}$ | $0.14 \pm 0.02^{c}$ |

CP : n = 6 independent extracts at the centre point (N° 4, 6, 9, 11,17 and 18)
n = 5 analyses per extract (absorbance) and n = 3 analyses per extract (TPC)
Rows with different superscripts are significantly different based on the analysis of variance ($p$<0.05)
